# METTL3-Mediated m6A Modification Regulates the Osteogenic Differentiation through LncRNA CUTALP in Periodontal Mesenchymal Stem Cells of Periodontitis Patients

**DOI:** 10.1155/2024/3361794

**Published:** 2024-01-19

**Authors:** Xin Chen, Yuan Qin, Xian Wang, Hao Lei, Xiaochen Zhang, Houzhuo Luo, Changgang Guo, Weifu Sun, Shishu Fang, Wen Qin, Zuolin Jin

**Affiliations:** ^1^State Key Laboratory of Military Stomatology and National Clinical Research Center for Oral Diseases and Shaanxi Clinical Research Center for Oral Diseases, Department of Orthodontics, School of Stomatology, Air Force Medical University, Xi'an 710032, China; ^2^Department of Dermatology, The First Affiliated Hospital of Xi'an Jiaotong University, Xi'an 730070, China

## Abstract

**Objective:**

Periodontitis is a chronic inflammatory disease that causes loss of periodontal support tissue. Our objective was to investigate the mechanism by which METTL3-mediated N6-methyladenosine modification regulates the osteogenic differentiation through lncRNA in periodontal mesenchymal stem cells in patients with periodontitis (pPDLSCs). *Material and Methods*. We carried out a series of experiments, including methylated RNA immunoprecipitation-PCR, quantitative real-time polymerase chain reaction, and western blotting. The expressions of alkaline phosphatase (ALP), Runx2, Col1, Runx2 protein level, ALP staining, and Alizarin red staining were used to demonstrate the degree of osteogenic differentiation.

**Results:**

We found that METTL3 was the most significantly differentially expressed methylation-related enzyme in pPDLSCs and promoted osteogenic differentiation of pPDLSCs. METTL3 regulated the stability and expression of lncRNA CUTALP, while lncRNA CUTALP promoted osteogenic differentiation of pPDLSCs by inhibiting miR-30b-3p. At different time points of osteogenic differentiation, lncRNA CUTALP expression was positively correlated with Runx2, while miR-30b-3p showed the opposite pattern. The attenuated osteogenic differentiation induced by *METTL3* knockdown was recovered by lncRNA CUTALP overexpression. The attenuated osteogenic differentiation induced by lncRNA CUTALP knockdown could be reversed by the miR-30b-3p inhibitor.

**Conclusions:**

In summary, METTL3/lncRNA CUTALP/miR-30b-3p/Runx2 is a regulatory network in the osteogenic differentiation of pPDLSCs.

## 1. Introduction

In recent years, periodontal regenerative medicine has attracted widespread attention in the field of stomatology [1]. Periodontal mesenchymal stem cells (PDLSCs) are the seed cells of oral tissue and are highly useful. Similar to mesenchymal stem cells (MSCs), PDLSCs show osteogenic differentiation, adipogenic differentiation, and chondrogenic differentiation [2]. Periodontitis is caused by multiple factors, resulting in varying degrees of loss of periodontal ligaments and alveolar bone [3]. In the periodontitis microenvironment, PDLSCs have been proven to act as a “double-edged sword,” showing anti-inflammatory and proinflammatory roles [4, 5]. Many scholars have demonstrated that PDLSCs in the periodontitis microenvironment have reduced osteogenic differentiation ability, leading to the further loss of alveolar bone [6]. The Wnt signaling pathway, NF-*κ*B signaling pathway, and MAPK signaling pathway were shown to be related to the role of PDLSCs in periodontitis [5–7]. Due to the stem cell function of PDLSCs, it is still necessary to explore the specific regulatory mechanism of osteogenic differentiation in periodontitis centered on PDLSCs.

Due to advances in sequencing technology, N6-methyladenosine (m6A), as one of the most representative epigenetic modifications, has been found on a variety of biological macromolecules and participates in the regulation of various biological processes [8, 9]. M6A methylation-related proteins include methyltransferase, demethylase, and RNA-binding proteins, which are named writers, erasers, and readers, respectively [10]. Methyltransferases such as METTL3 and METTL14 can form m6A modifications, while demethylases such as FTO and ALKBH5 can erase m6A modifications. RNA binding proteins, including YTHDF1/2/3 and IGF2BP1/2/3, can recognize m6A modifications [10, 11]. In recent years, researchers have focused on m6A modification and stem cells to determine the regulatory network between the two. Knockdown of *METTL3* and *METTL14* was shown to inhibit the self-renewal of mouse embryonic stem cells (mESCs) [12]. M6A modification was shown to mediate lincRNA1281 in mESC differentiation [13]. Accumulating evidence indicates that the generation and maintenance of cancer stem cells require the involvement of m6A modification, as does the genesis and development of cancer [14]. In the field of stomatology, the research of m6A methylation also involves various aspects. Chen et al. [15] found that METTL3-mediated ALDH m6A methylation regulated the malignant behavior of BMI1+ HNSCC stem cells. Moreover, METTL14 promoted oral squamous cell carcinoma progression by regulating the mRNA and m6A levels of *CALD1* [16]. Pan et al. [17] found that METTL3-enhanced dentinogenesis differentiation of dental pulp stem cells via increasing *GDF6* and *STC1* mRNA stability. Tian et al. [18] found that *Alkbh5* overexpression in the mouse dental papilla cell line mDPC6T promoted odontoblastic differentiation. In addition, a study demonstrated that m6A methylation is activated in PDLSCs after LPS treatment [19]. Another study indicated that the process by which LPS inhibits the osteogenic differentiation of the human periodontal ligament (HPDL) may be affected by DNA hypermethylation [20]. Based on the above studies, we speculated that the attenuated osteogenic ability of periodontal mesenchymal stem cells in patients with periodontitis (pPDLSCs) was related to m6A modification.

In recent years, scientists have focused on m6A modification in ncRNAs and have found that m6A modification regulates the expression and structure of ncRNA to affect various physiological activities and disease progression [21]. Zuo et al. [22] found that m6A modification promoted the upregulation of LINC00958, thus promoting lipogenesis. Song et al. [23] demonstrated that METTL3-mediated m6A modification activates the MAPK pathway by regulating lncRNA RP11-44N12.5, thereby promoting osteogenic differentiation of human adipose-derived stem cells (hASCs). Previously, our group proved that both lncRNA TWIST1 and lncRNA-POIR have regulatory roles in pPDLSCs osteogenic differentiation [24, 25]. Therefore, we speculated that the regulatory mechanism of lncRNAs in pPDLSCs is related to their m6A modification.

LncRNAs are widely distributed in cells, including the nucleus, cytoplasm, organelles, and cell periphery [26]. The functions of lncRNAs are closely related to their subcellular localization [27, 28]. LncRNAs located in the cytoplasm have higher stability [29] and are involved in signal transduction, translation, and regulation of posttranscriptional gene expression [26]. For example, many lncRNAs located in the cytoplasm act as sponges to adsorb miRNAs, thereby inhibiting the regulation of target genes by miRNAs [30]. Many studies have confirmed that the process of osteogenic differentiation in various stem cells is regulated by the lncRNA–miRNA–mRNA network [31–35]. In human bone marrow-derived mesenchymal stem cells (hBMSCs), lncRNA MALAT1 regulates Osterix (Osx) expression to promote osteogenic differentiation by binding and inhibiting miR-143 [36]. Peng et al. [37] found that lncRNA ANCR regulated Notch2 expression to inhibit osteogenic differentiation in PDLSCs by inhibiting miR-758. Our group previously found through microarray analysis that periodontitis osteogenic lncRNA (lncRNA POIR) was downregulated in pPDLSCs and upregulated FoxO1 by inhibiting miR-182, thereby activating the Wnt pathway [25]. Although the regulation of the lncRNA–miRNA–mRNA network has been widely studied, whether this regulatory mode is affected by m6A modification needs to be further verified.

Previous studies on the reduced osteogenic ability of pPDLSCs have focused on the lncRNA‒miRNA–mRNA regulatory network, indicating their limitations. Therefore, we wondered whether this classical regulatory network is also affected by other factors, such as m6A modification of lncRNAs. To test this hypothesis, we conducted microarray analysis between human periodontal mesenchymal stem cells (hPDLSCs) and pPDLSCs to screen for differentially methylated lncRNAs and explored the effect of the m6A-related protein METTL3 on the osteogenic differentiation of pPDLSCs in vitro. We identified the key differentially methylated lncRNAs in pPDLSCs from the microarray, verified them by methylated RNA immunoprecipitation-PCR (MeRIP-qPCR), and discussed their role in the osteogenic differentiation of pPDLSCs. Moreover, the regulatory effect of METTL3 and lncRNAs was studied, and the downstream miRNAs and target genes of lncRNAs were identified. The study was to explore the effect of METTL3-mediated m6A modification on the lncRNA‒miRNA–mRNA regulatory network in the osteogenic differentiation of pPDLSCs.

## 2. Materials and Methods

### 2.1. Samples and Cell Culture

Both hPDLSCs and pPDLSCs were cultured from 8 patients aged 31–40 years [38] (patient clinical information is provided in Table S1). hPDLSCs were routinely extracted from premolars or third molars for orthodontic reasons. The teeth for pPDLSC extraction were from individuals diagnosed with periodontitis. These teeth had a 2/3 loss of alveolar bone and at least one periodontal pocket more than 5 mm deep, and other systemic and surgical history was excluded [25]. All samples were collected from the Department of Maxillofacial Surgery, Stomatological Hospital of the Fourth Military Medical University, and informed consent was obtained from the subjects. The study was approved by the Ethical Committee of the hospital (licence number: IRB-Rev-2020005). The middle 1/3 healthy and inflammatory tissues of roots were collected and treated with collagenase I (0.66 mg/ml; Sigma, St Louis, Missouri, USA) for 30 min. Cells were then incubated in the minimum medium required (*α*-MEM; 10% fetal bovine serum, 0.292 mg/ml glutamine (Invitrogen, Carlsbad, CA, USA), 100 U/ml penicillin and 100 mg/ml streptomycin (Gibco BRL)), The cells were incubated at 37°C in a humidified atmosphere of 5% CO_2_ and 95% air until they climbed out. PDLSCs from the same source were used in each experiment, and third-generation cells were used in all experiments.

### 2.2. Flow Cytometry

hPDLSCs and pPDLSCs were incubated with antibodies against CD73 (BioLegend, Cat# 344017, RRID: AB_2564561), CD90 (BioLegend, Cat# 328106, RRID: AB_893447), CD105 (BioLegend, Cat# 323214, RRID: AB_2293527), CD146 (BioLegend, Cat# 361036, RRID: AB_2814306), CD11b (BioLegend, Cat# 379902, RRID: AB_2910448), CD19 (BioLegend, Cat# 302202, RRID: AB_314232), CD31 (BioLegend, Cat# 303102, RRID: AB_314328), CD34 (BioLegend, Cat# 343502, RRID: AB_1731898), CD45 (BioLegend, Cat# 361902, RRID: AB_2563177), and HLA-DR (BioLegend, Cat# 327002, RRID: AB_893582) according to the manufacturer's instructions [39, 40].

### 2.3. Colony-Forming Assays

A total of 500 hPDLSCs and pPDLSCs were seeded in 10 cm dishes, and the cell masses were observed under a microscope after about 2 weeks of culture. The samples were fixed with 4% paraformaldehyde solution for 30 min and stained with crystal violet solution (MedChem Express, China). After washing 3–5 times with PBS, the number of colonies in each Petri dish was counted under a microscope to calculate the rate of colony formation.

### 2.4. Cell Proliferation Assay

A total of 100 hPDLSCs or pPDLSCs were seeded in 96-well plates and cultured with the minimum necessary medium. Cell Counting Kit-8 (CCK-8; NCM Biotech, Zhejiang, China) was used. On days 1, 2, 3, 4, 5, 6, 7, and 8, a microplate reader (Cytation 5; BioTek, VT, USA) was used to detect the absorbance at 450 nm.

### 2.5. Osteogenic and Adipogenic Differentiation

Cells were incubated with OriCell® human bone marrow MSC osteogenic differentiation medium (HUXMX-90021) for 21 days, and the solution was changed every 3 days. Cells were fixed with 4% paraformaldehyde and stained with alizarin red. Similarly, cells were incubated with OriCell® human bone marrow MSC adipogenic differentiation medium (HUXMX-90031) for 21 days. Fixed with 4% paraformaldehyde and stained with oil red O solution. These experiments were repeated three times.

### 2.6. Alizarin Red Staining (ARS) and Alkaline Phosphatase (ALP) Staining

ALP staining was performed 7 days after osteogenic induction using the 5-bromo-4-chloro-3-indole phosphate/nitroblue-tetrazolium ALP chromogenic Kit (Beyotime, Shanghai, China). ARS was performed 21 days after induction of osteogenesis. Fixed with 4% paraformaldehyde for 30 min before staining.

### 2.7. Microarray Screening and Analysis

Total RNA was quantified in hPDLSC samples and pPDLSC samples using a NanoDrop ND-1000. Sample preparation assays and microarray hybridization were performed according to Arraystar's standard protocols. RNA was immunoprecipitated using m6A antibody. IP was eluted from sediment magnetic beads in the presence of m6A-modified RNA, and Sup was recovered from the supernatant unmodified RNA. IP and Sup RNAs were labeled as cRNAs with Cy5 and Cy3 using an Arraystar Super RNA Labeling Kit. These cRNAs were combined and hybridized onto an Arraystar human mRNA & lncRNA epitope microarray (8x60K, Arraystar). An Agilent G2505C scanner was used to scan the array in two-colour channels. The obtained array images were analyzed using Agilent feature extraction software (version 11.0.1.1). The m6A methylation level for a transcript was calculated as the percentage of modified RNA (%Modified) in all RNAs based on the IP (Cy5-labelled) and Sup (Cy3-labelled) normalized intensities:

(1) $$\begin{array}{c} \text{\% Modified}=\frac{\text{Modified }RNA}{\text{Total }RNA}=\frac{IP}{IP+Sup}=\frac{{IP}_{\text{Cy5 normalized intensity}}}{{IP}_{\text{Cy5 normalized intensity}}+{Sup}_{\text{Cy3 normalized intensity}}}. \end{array}$$

The “m6A quantity” was calculated for the m6A methylation amount of each transcript based on the IP (Cy5-labeled) normalized intensities:

(2) $$\begin{array}{c} \text{Sample A }m6A\text{ quantity}=\text{Sample A }{IP}_{\text{Cy5 normalized intensity}}. \end{array}$$

According to the “m6A quantity,” we screened with a fold change >2 or <0.5 and statistical significance (*P* ≤ 0.05) threshold and identified the top three hypermethylated lncRNAs and the top three hypomethylated lncRNAs in pPDLSCs. The details are described in Section 3.

### 2.8. Quantitative Real-Time Polymerase Chain Reaction (qRT-PCR)

Total RNA was extracted using TRIzol reagent (Accurate Biotechnology, Hunan, China) and converted into cDNA (Evo M-MLV RT Premix for qPCR; Accurate Biotechnology). The miRNA RNAs were converted into cDNA using a miRNA 1st strand cDNA synthesis kit (Accurate Biotechnology, Hunan, China). *β*-Actin and U6 were used as reference controls for mRNAs, lncRNAs, and miRNAs. Primers were designed by Accurate Biotechnology (Hunan, China) (Table S2). qPCR was performed using the SYBR® Green Premix Pro Taq HS qPCR Kit (Accurate Biotechnology, Hunan, China) and the Applied Biosystems 7500 Real-Time PCR Detection System. The data were analyzed using the 2^−*ΔΔ*Ct^ (Livak) relative expression method. All experiments were repeated three times.

### 2.9. Western Blotting

Total protein was first extracted using Western and IP cell lysis buffer (Beyotime, P0013). BCA protein assay kit (Zhonghui Hecai, Shaanxi, China) was used to determine the protein concentration obtained, and 30 *μ*g of total protein was obtained. Then, electrophoresis was performed according to previous studies [25], and PVDF membranes (Millipore, Billerica, Massachusetts, USA) were used for protein transformation. Subsequently, primary antibodies against METTL3 (monoclonal) (1 : 1,000, E3F2A, Cell Signaling), Runx2 (monoclonal) (1 : 1,000, ab236639, Abcam), and GAPDH (monoclonal) (1 : 1,000, D11H16, Cell Signaling) were incubated with membranes. TBST is used to wash the membranes, and then incubated membranes with the corresponding secondary antibody (polyclonal) (1 : 10,000, Invitrogen, USA) for 1 hr. ImageJ software (V1.8.0) was used for quantitative analysis.

### 2.10. Transfection of MiRNA Mimic and Inhibitor

Transfection of miRNA mimic and inhibitor (GenePharma, Shanghai, China) was performed according to the instructions. Six microliters of mimic/inhibitor and 5 *µ*l of Gp Transfect Mate (GenePharma, Shanghai, China) were each added to two 200 *µ*l aliquots of *α*-medium. The above two fluids were mixed and left at rest for 30 min and then added to 1.6 ml of minimum necessary medium to transfect the cells. The supplementary data shows the specific sequences.

### 2.11. Construction and Transduction of Lentivirus

Lentiviral production was performed by GenePharma (Shanghai, China). LV-NC5 and LV-NC3 were the negative controls for the overexpression and knockdown vectors, respectively. Lentiviral vectors were added to the minimum necessary medium according to the instructions, and polybrene (GenePharma, Shanghai, China) was cotransfected to enhance transfection efficiency. The sequences of METTL3 and lncRNA are described in the supplementary data. P3 hPDLSCs were inoculated into 96-well plates and infected with infection multiples (MOI) of 0, 30, 60, and 90. The medium was changed 24 hr later, and the cells were observed under an inverted fluorescence microscope 72 hr later. The expression of green fluorescent protein (GFP) in hPDLSCs was observed to determine the optimal MOI value, and then formal experiments were conducted according to the above methods, and the optimal MOI was used. Seventy-two hours later, the expression of GFP was observed under an inverted fluorescence microscope, and the transfection efficiency was measured (Figures S1 and S2). In addition, qRT-PCR and western blotting were used to detect transfection efficiency.

### 2.12. MeRIP-qPCR

MeRIP-qPCR was based on a previous method [41]. About 1–3 *μ*g of total RNA was extracted, and the concentration and quality of RNA were assessed by NanoDrop spectrophotometer and denaturing agarose gel electrophoresis. M6A-modified mRNA was immunoprecipitated with an anti-m6A antibody (Synaptic System, #202003) after 10% of the fragmented mRNA was saved as input. A suspension of 20 *μ*l Dynabeads and M280 goat antirabbit IgG (Invitrogen, 11203D) was incubated for 2 hr at 4°C. The enriched RNA was obtained in the eluent and then detected by qRT-PCR.

### 2.13. Fluorescence In Situ Hybridization (FISH)

Subcellular distribution of lncRNA CUTALP was assessed using a FISH kit according to the manufacturer's instructions (GenePharma, Shanghai, China). In brief, pPDLSCs were fixed in 4% paraformaldehyde and hybridized with a 10 *μ*M Cy3-labeled lncRNA CUTALP probe in a hybridization buffer. Cells were washed with saline sodium citrate and counterstained with DAPI. Images were obtained using a fluorescence microscope.

### 2.14. RNA-Binding Protein Immunoprecipitation (RIP) Assay

A RIP assay detection kit (Merck Millipore) was used according to the manufacturer's instructions. RIP lysis buffer was prepared for cell lysis with 100 *μ*l of RIP lysis buffer, 0.5 *μ*l of protease inhibitor cocktail, and 0.25 *μ*l of RNase inhibitor. Anti-METTL3 (1 : 50, E3F2A, Cell Signaling) was added to the resuspended beads, and the mixture was incubated for 30 min at room temperature. RIP immunoprecipitation buffer was prepared for RNA-binding protein immunoprecipitation with 860 *μ*l of RIP wash buffer, 35 *μ*l of 0.5 M EDTA, and 5 *μ*l of RNase inhibitor. The RNA bound to the beads was purified and then reverse-transcribed into cDNA for qRT-PCR.

### 2.15. RNA Stability

Each well of a six-well plate was seeded with 2 × 10^5^ cells. After 2 days of incubation, 2 *µ*l of 5 *μ*g/ml actinomycin D (ACT-D, Catalogue #A9415, Sigma, St. Louis, MO, USA) and 2 ml of minimal medium were added to each well. Cells were collected after adding actinomycin D at 0, 2, 4, 6, and 8 hr.

### 2.16. Dual-Luciferase Reporter Assay

The predicted binding sites of lncRNA and miRNA were cloned into the GPmiRGLO dual-luciferase reporter vector (GenePharma, Shanghai, China). Thus, wild-type GPmiRGLO-lncRNA and mutated GPmiRGLO-lncRNA were constructed. Then, 293 T cells were transiently cotransfected with wild/mutated GPmiRGLO-lncRNA with miRNA mimic or miR-NC. Detection and analysis were performed using a dual-luciferase reporter detection system (Tecan M1000, Austria, Switzerland).

### 2.17. Immunofluorescence (IF) Analysis

pPDLSCs were fixed in 4% paraformaldehyde, permeated in 0.1% Triton X-100, and blocked with 5% BSA. Then, cells were sequentially incubated with primary antibody (Anti-METTL3, cat no. 67733-1-Ig, proteintech) and Alexa Fluorescein (FITC)-conjugated Affinipure Goat anti-mouse IgG (H + L) (Cat No. SA00003-1), followed by DAPI staining. Images were obtained using a fluorescence microscope.

### 2.18. Statistical Analysis

GraphPad Prism 8.0.1 was used for statistical analysis and graphic production. Data are expressed as the mean ± standard deviation. Student's *t*-test was used for data analysis between two samples, and one-way ANOVA was used for multiple samples. Significance levels were determined by ( ^*∗*^) *P*  < 0.05, ( ^*∗∗*^) *P*  < 0.01, ( ^*∗∗∗*^) *P*  < 0.001, and ( ^*∗∗∗∗*^) *P*  < 0.0001.

## 3. Results

### 3.1. METTL3 Promoted Osteogenic Differentiation of hPDLSCs and pPDLSCs In Vitro

**Figure 1:**
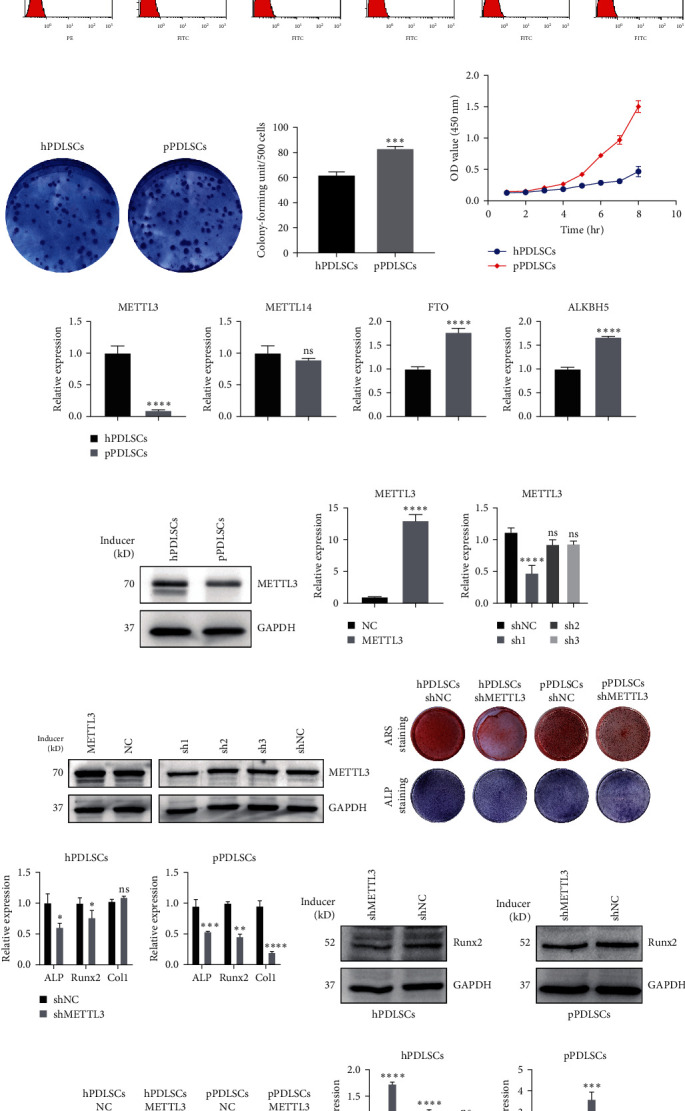
Cell identification and METTL3 promoted osteogenic differentiation of hPDLSCs and pPDLSCs: (a) surface makers of hPDLSCs and pPDLSCs were identified by flow cytometry; (b) clone formation assays of hPDLSCs and pPDLSCs; (c) the quantitative analysis diagram for clone formation assays; (d) cell proliferation assays of hPDLSCs and pPDLSCs; (e) the expressions of methyltransferase (METTL3 & METTL14) and demethylases (ALKBH5 and FTO) in hPDLSCs and pPDLSCs; (f) the protein levels of METTL3 in hPDLSCs and pPDLSCs; (g and h) transfection efficiency of METTL3 was detected by qRT-PCR and western blotting; (i) osteogenic differentiation was detected by ARS staining and ALP staining at 21 and 7 days of osteogenic induction after METTL3 knockdown; (j) the mRNA levels of ALP, Runx2, and Col1 were detected at 7 days of osteogenic induction after METTL3 knockdown; (k) the protein level of Runx2 was determined by western blotting at 7 days of osteogenic induction after METTL3 knockdown; (l) osteogenic differentiation was detected by ARS staining and ALP staining at 21 and 7 days of osteogenic induction after METTL3 overexpression; (m) the mRNA levels of ALP, Runx2, and Col1 were detected at 7 days of osteogenic induction after METTL3 overexpression; (n) the protein level of Runx2 was determined by western blotting at 7 days of osteogenic induction after METTL3 overexpression. Significance levels were determined by ( ^*∗*^) *P*  < 0.05, ( ^*∗∗*^) *P*  < 0.01, ( ^*∗∗∗*^) *P*  < 0.001, and ( ^*∗∗∗∗*^) *P*  < 0.0001.

Under an inverted microscope, P3 hPDLSCs and pPDLSCs showed long spindle-shaped morphology and adherent growth. The cell morphology of pPDLSCs was more elongated than that of hPDLSCs (Figure S3). First, flow cytometry was used to identify surface markers of hPDLSCs and pPDLSCs. The results were the same in the two cell lines: CD73, CD90, CD105, and CD146 were positive, while CD11b, CD19, CD31, CD34, CD45, and HLA-DR were negative (Figure 1(a)). Clone formation and cell proliferation assays showed that pPDLSCs had higher proliferation than hPDLSCs (Figure 1(b)–1(d)). Previously, microarray analysis indicated that the total m6A modification of lncRNAs in pPDLSCs was lower than that in hPDLSCs [42], suggesting that m6A modification might be involved in the development of periodontitis. We detected the expression of several m6A-related enzymes and found that the expression of methyltransferase (METTL3) was lower in pPDLSCs than in hPDLSCs, while the expressions of demethylases (ALKBH5 and FTO) were higher in pPDLSCs than in hPDLSCs. However, the expression of methyltransferase (METTL14) had no significance (Figure 1(e)). The expression of METTL3 changed most significantly, and its protein content changed considerably (Figure 1(f)). To further explore the role of METTL3 in the osteogenic differentiation of PDLSCs, we performed lentivirus-mediated overexpression and knockdown of METTL3 in PDLSCs and observed the effects on osteogenesis. We assessed the transfection efficiency by qRT-PCR (Figure 1(g)) and western blotting in pPDLSCs (Figure 1(h)) and selected the most efficient construct for transfection. ARS staining of hPDLSCs and pPDLSCs showed decreased mineralized bone matrix formation after METTL3 knockdown and ALP staining showed decreased ALP decomposition products in the cells (Figure 1(i)). Quantitative analysis of ARS and ALP staining showed the same results (Figure S4). Moreover, the mRNA levels of ALP, Runx2, and Col1 were almost decreased after 7 days of osteogenic induction (Figure 1(j)). Similarly, the protein level of Runx2 was decreased (Figure 1(k)). In conclusion, METTL3 knockdown inhibited the osteogenic differentiation of hPDLSCs and pPDLSCs. In addition, we obtained the opposite results after overexpression of METTL3. ARS and ALP staining showed increased mineralized bone matrix formation after overexpression of METTL3 (Figure 1(l)), and quantitative analysis of ARS and ALP staining showed the same results (Figure S5). qRT-PCR showed almost increased mRNA levels of ALP, Runx2 and Col1 after 7 days of osteogenic induction (Figure 1(m)). Similarly, the protein content of Runx2 was increased (Figure 1(n)). As mentioned above, we demonstrated that METTL3 is a crucial regulator of the osteogenic differentiation of hPDLSCs and pPDLSCs.

### 3.2. METTL3 Promoted Osteogenic Differentiation by Promoting the Expression and Stability of LncRNA CUTALP

**Figure 2:**
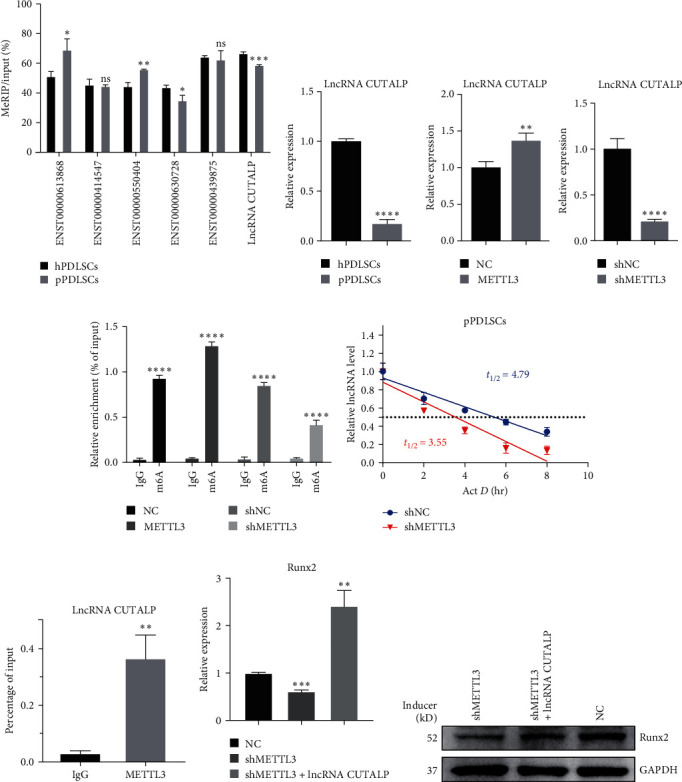
METTL3 promoted osteogenic differentiation by promoting the expression and stability of lncRNA CUTALP: (a) MeRIP-qPCR was used to detect the six m6A-modified lncRNAs; (b) the expression levels of lncRNA CUTALP in hPDLSCs and pPDLSCs were detected by qRT-PCR; (c and d) the expression and methylation modification levels of lncRNA CUTALP after METTL3 overexpression and knockdown in pPDLSCs were detected by qRT-PCR and MeRIP-qPCR; (e) the RNA decay rate assay was detected the half-life of lncRNA CUTALP after METTL3 knockdown; (f) the RIP-qPCR was carried to confirm the binding between METTL3 and lncRNA CUTALP; (g and h) qRT-PCR and western blotting showed that the inhibition of osteogenic markers caused by METTL3 knockdown in pPDLSCs would be recovered by lncRNA CUTALP overexpression. Significance levels were determined by ( ^*∗*^) *P*  < 0.05, ( ^*∗∗*^) *P*  < 0.01, ( ^*∗∗∗*^) *P*  < 0.001, and ( ^*∗∗∗∗*^) *P*  < 0.0001.

We screened the top three hypermethylated lncRNAs and the top three hypomethylated lncRNAs according to the methods mentioned above, and MeRIP-qPCR was used to detect the six m6A-modified lncRNAs. Then, we identified and named a new lncRNA, lncRNA CUTALP (Transcript ID: NR_024408), which had the most significant change by MeRIP-qPCR (Figure 2(a)). Some of the results were consistent with the m6A quantity (Table 1). qRT-PCR showed that the expression level of lncRNA CUTALP in pPDLSCs was significantly lower than that in hPDLSCs (Figure 2(b)). METTL3 is a methyltransferase that is necessary for the formation of methylation. Consequently, we speculated whether it could affect the expression of lncRNA CUTALP by regulating its methylation. We found that in pPDLSCs, the expression and methylation modification level of lncRNA CUTALP increased after *METTL3* overexpression, while the expression and methylation modification level of lncRNA CUTALP decreased after *METTL3* knockdown (Figures 2(c) and 2(d)). Moreover, the RNA decay rate assay demonstrated that lncRNA CUTALP half-life was significantly shortened upon *METTL3* knockdown (Figure 2(e)). In addition, the binding between METTL3 and lncRNA CUTALP was confirmed by RIP-qPCR (Figure 2(f)), and we found that the inhibition of the mRNA and protein levels of osteogenic markers by *METTL3* knockdown could be restored by overexpression of the lncRNA CUTALP (Figures 2(g) and 2(h)). These results confirmed that METTL3 promoted osteogenesis by promoting the expression and stability of lncRNA CUTALP.

### 3.3. LncRNA CUTALP Promoted Osteogenic Differentiation of hPDLSCs and pPDLSCs In Vitro

**Figure 3:**
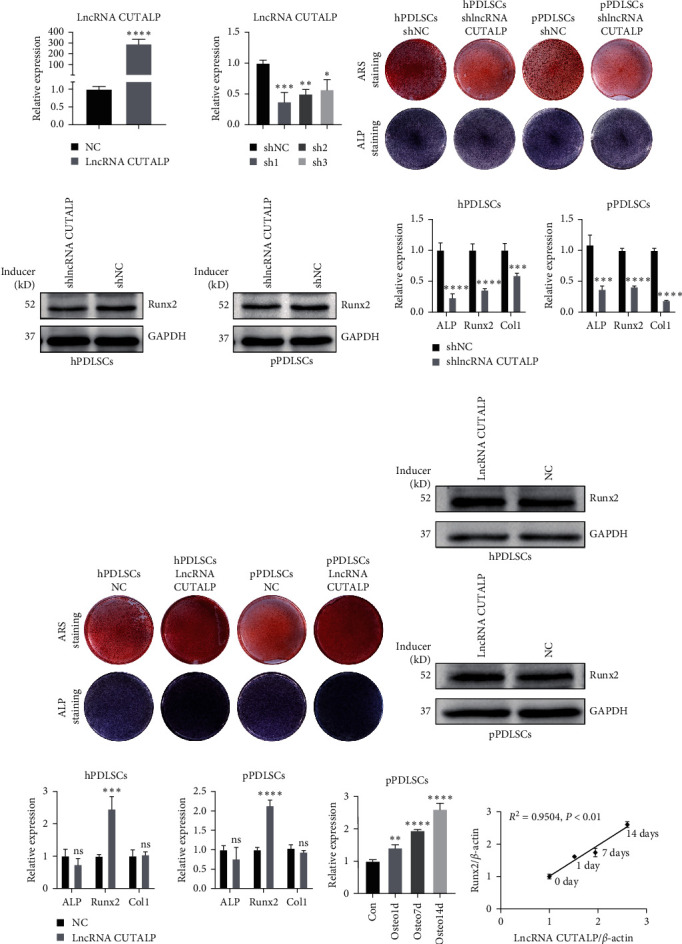
LncRNA CUTALP promoted osteogenic differentiation of hPDLSCs and pPDLSCs: (a) transfection efficiency of lncRNA CUTALP was detected by qRT-PCR; (b) osteogenic differentiation was detected by ARS staining and ALP staining at 21 and 7 days of osteogenic induction after lncRNA CUTALP knockdown; (c) the protein level of Runx2 was determined by western blotting at 7 days of osteogenic induction after lncRNA CUTALP knockdown; (d) the mRNA levels of ALP, Runx2, and Col1 were detected at 7 days of osteogenic induction after lncRNA CUTALP knockdown; (e) osteogenic differentiation was detected by ARS staining and ALP staining at 21 and 7 days of osteogenic induction after lncRNA CUTALP overexpression; (f) the mRNA levels of ALP, Runx2, and Col1 were detected at 7 days of osteogenic induction after lncRNA CUTALP overexpression; (g) the protein level of Runx2 was determined by western blotting at 7 days of osteogenic induction after lncRNA CUTALP overexpression; (h) qRT-PCR was used to detect the expression changes of lncRNA CUTALP with osteogenic induction; (i) correlation analysis between lncRNA CUTALP levels and Runx2 levels in pPDLSCs 0, 1, 7, and 14 days after osteogenic induction. Significance levels were determined by ( ^*∗*^) *P*  < 0.05, ( ^*∗∗*^) *P*  < 0.01, ( ^*∗∗∗*^) *P*  < 0.001, and ( ^*∗∗∗∗*^) *P*  < 0.0001.

To further understand the role of lncRNA CUTALP in the osteogenic differentiation of PDLSCs, we constructed a lncRNA CUTALP overexpression and knockdown vector to transfect PDLSCs and conducted experiments. We evaluated the transfection efficiency by qRT-PCR in pPDLSCs and selected the most efficient construct for transfection (Figure 3(a)). ARS staining of hPDLSCs and pPDLSCs showed decreased mineralized bone matrix formation after lncRNA CUTALP knockdown, and ALP staining showed decreased ALP decomposition products in the cells (Figure 3(b)). Quantitative analysis of ARS and ALP staining showed the same results (Figure S6). Similarly, the protein level of Runx2 and the mRNA levels of ALP, Runx2, and Col1 were decreased after 7 days of osteogenic induction (Figures 3(c) and 3(d)). These results demonstrated that lncRNA CUTALP knockdown inhibits the osteogenic differentiation of hPDLSCs and pPDLSCs. In contrast, ARS staining, ALP staining, the protein level of Runx2, and the expression level of Runx2 were increased, but the expression levels of ALP and Col1 were not significantly changed after lncRNA CUTALP overexpression (Figure 3(e)–3(g)). Quantitative analysis of ARS and ALP staining showed the same results (Figure S7). qRT-PCR indicated that the expression of lncRNA CUTALP was increased with osteogenic induction (Figure 3(h)). We also found a highly positive correlation between lncRNA CUTALP and Runx2 expression in pPDLSCs at 0, 1, 7, and 14 days after osteogenic induction (Figure 3(i)). In conclusion, lncRNA CUTALP is an important regulatory factor promoting osteogenic differentiation of PDLSCs.

### 3.4. LncRNA CUTALP Regulated the Osteogenic Differentiation of pPDLSCs via Competitively Binding MiR-30b-3p

**Figure 4:**
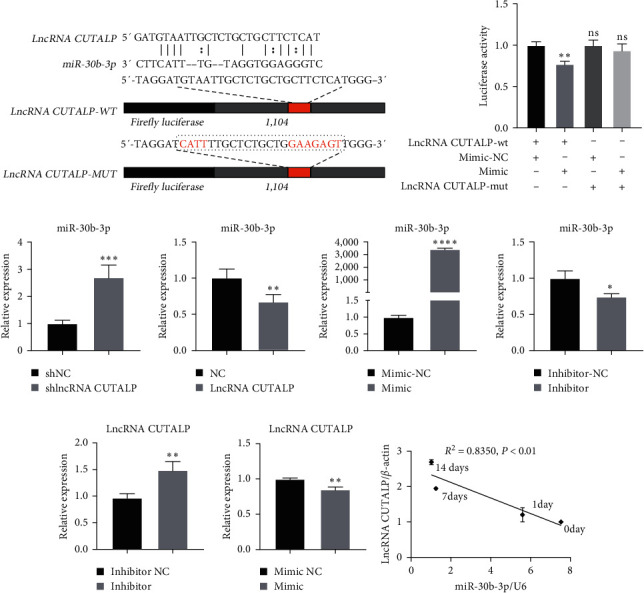
LncRNA CUTALP regulated the osteogenic differentiation of pPDLSCs via competitively binding miR-30b-3p: (a) schematic diagram and sequences of miR-30b-3p predicted target site in lncRNA CUTALP; (b) dual-luciferase reporter assays were performed to measure the luciferase activity in 293T cells; (c) qRT-PCR was used to detect the expression level of miR-30b-3p after lncRNA CUTALP knockdown and overexpression; (d) transfection efficiency of miR-30b-3p mimic and inhibitor was detected by qRT-PCR; (e) the expression levels of lncRNA CUTALP after transfection with miR-30b-3p mimic and inhibitor were detected by qRT-PCR; (f) correlation analysis between miR-30b-3p levels and lncRNA CUTALP levels in pPDLSCs 0, 1, 7, and 14 days after osteogenic induction. Significance levels were determined by ( ^*∗*^) *P*  < 0.05, ( ^*∗∗*^) *P*  < 0.01, ( ^*∗∗∗*^) *P*  < 0.001, and ( ^*∗∗∗∗*^) *P*  < 0.0001.

To determine the specific regulatory mechanism of lncRNA CUTALP on osteogenic differentiation, we performed bioinformatics analysis and prediction. Based on the results of the lncLocator analysis, lncRNA CUTALP is mostly located in the cytoplasm. Moreover, miR-30b-3p was predicted to directly bind to lncRNA CUTALP via MicroInspector online software (http://bioinfo.uni-plovdiv.bg/microinspector). To verify the predictions, we conducted a series of experiments. First, we found that the lncRNA CUTALP sequence contains a unit that binds complementarily to miR-30b-3p (Figure 4(a)). A dual-luciferase reporter assay showed that luciferase activity was significantly decreased in the miR-30b-3p mimic and lncRNA CUTALP-wt cotransfection groups, while there was no change in other groups (Figure 4(b)). Also, the expression of miR-30b-3p increased after lncRNA CUTALP knockdown, while it decreased after overexpression of lncRNA CUTALP (Figure 4(c)). For further study, we constructed miR-30b-3p mimics and inhibitors and verified the transfection efficiency by qRT-PCR (Figure 4(d)). The increase in lncRNA CUTALP expression was caused by the miR-30b-3p inhibitor in pPDLSCs, while the decrease was made by the miR-30b-3p mimic (Figure 4(e)). We also found that lncRNA CUTALP and miR-30b-3p expression in pPDLSCs was highly negatively correlated at 0, 1, 7, and 14 days after osteogenic induction (Figure 4(f)). Overall, these results indicated that lncRNA CUTALP and miR-30b-3p negatively regulate each other.

### 3.5. MiR-30b-3p Inhibited Osteogenic Differentiation of pPDLSCs In Vitro

**Figure 5:**
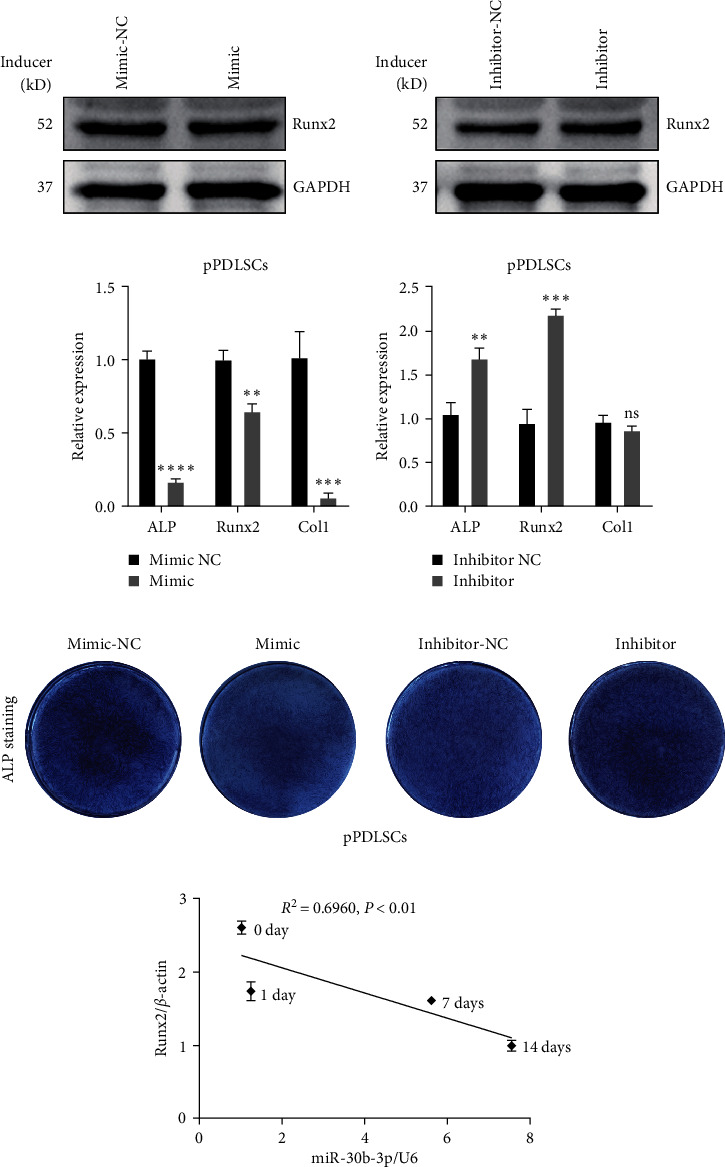
MiR-30b-3p inhibited osteogenic differentiation of pPDLSCs: (a) the protein level of Runx2 was determined by western blotting at 7 days of osteogenic induction transfection with miR-30b-3p mimic and inhibitor; (b) the mRNA levels of ALP, Runx2, and Col1 were detected at 7 days of osteogenic induction after transfection with miR-30b-3p mimic and inhibitor; (c) osteogenic differentiation was detected by ALP staining at 7 days of osteogenic induction after transfection with miR-30b-3p mimic and inhibitor; (d) correlation analysis between miR-30b-3p levels and Runx2 levels in pPDLSCs 0, 1, 7, and 14 days after osteogenic induction. Significance levels were determined by ( ^*∗∗*^) *P*  < 0.01, ( ^*∗∗∗*^) *P*  < 0.001, and ( ^*∗∗∗∗*^) *P*  < 0.0001.

Based on previous studies on the miR-30 family, we focused on whether miR-30b-3p is involved in and regulates the osteogenic differentiation of pPDLSCs. After pPDLSCs were transfected with miR-30b-3p mimic, the protein level of Runx2 was lower than that in the mimic-NC transfected cells for 7 days after osteogenic induction, while the opposite results were obtained after transfection with miR-30b-3p inhibitor (Figure 5(a)). Subsequently, after 7 days of osteogenic induction, qRT-PCR showed that the miR-30b-3p mimic inhibited the mRNA expression of ALP, Runx2, and Col1, and the miR-30b-3p inhibitor promoted the mRNA expression of ALP and Runx2 (Figure 5(b)). ALP staining showed decreased ALP decomposition products after transfection with the mimic, and the opposite was observed after transfection with the inhibitor in pPDLSCs (Figure 5(c)). Quantitative analysis of ALP staining showed the same results (Figure S8). We also found that miR-30b-3p and Runx2 expression in pPDLSCs was highly negatively correlated at 0, 1, 7, and 14 days after osteogenic induction (Figure 5(d)). In conclusion, we demonstrated that miR-30b-3p inhibits the osteogenic differentiation of pPDLSCs, which is opposite to the function of lncRNA CUTALP.

### 3.6. LncRNA CUTALP Modulated Runx2 through Regulation of MiR-30b-3p

**Figure 6:**
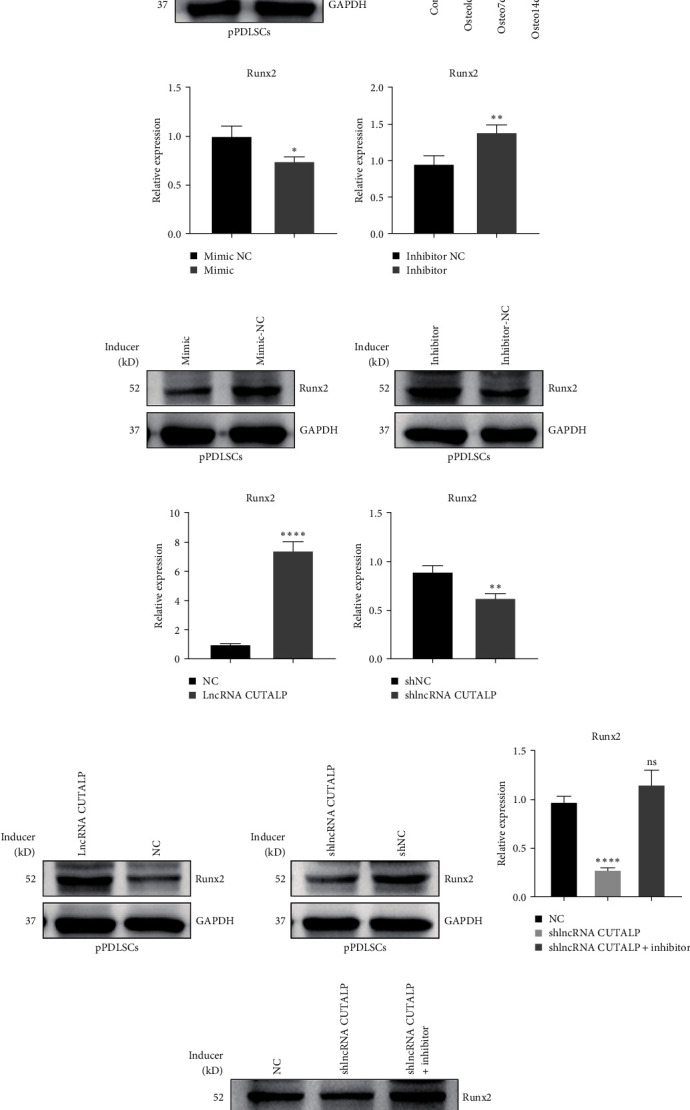
LncRNA CUTALP modulated Runx2 through regulation of miR-30b-3p: (a) the protein level of Runx2 was detected by western blotting after osteogenic differentiation in pPDLSCs; (b) qRT-PCR was used to detect the expression changes of Runx2 with osteogenic induction; (c) the mRNA levels of Runx2 were detected by qRT-PCR after transfection with miR-30b-3p mimic and inhibitor; (d) the protein levels of Runx2 were detected by western blotting after transfection with miR-30b-3p mimic and inhibitor; (e) the mRNA levels of Runx2 were detected by qRT-PCR after lncRNA CUTALP overexpression and knockdown; (f) the protein levels of Runx2 were detected by western blotting after ncRNA CUTALP overexpression and knockdown; (g and h) qRT-PCR and western blotting showed that the inhibition of osteogenic markers caused by lncRNA CUTALP knockdown in pPDLSCs would be recovered by miR-30b-3p inhibitor. Significance levels were determined by ( ^*∗*^) *P*  < 0.05, ( ^*∗∗*^) *P*  < 0.01, ( ^*∗∗∗*^) *P*  < 0.001, and ( ^*∗∗∗∗*^) *P*  < 0.0001.

**Figure 7:**
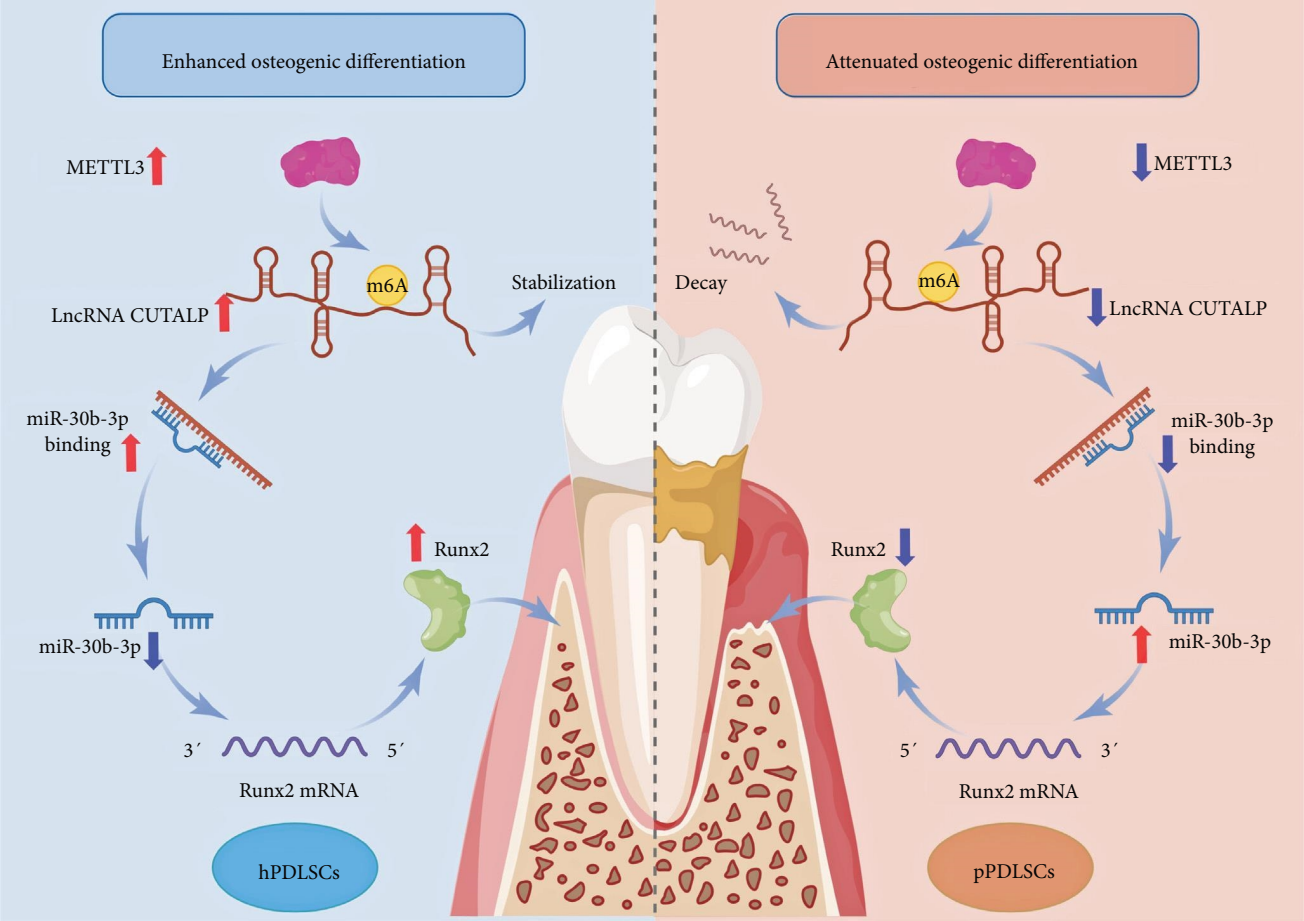
The expression of m6A-related protein METTL3 in pPDLSCs was lower than that in hPDLSCs. METTL3 promoted the expression and stability of downstream lncRNA CUTALP by regulating m6A modification. LncRNA CUTALP increased the expression of Runx2 through competitive binding of miR-30b-3p, thereby promoting osteogenic differentiation. METTL3/lncRNA CUTALP/miR-30b-3p/Runx2 is a regulatory network for osteogenic differentiation of pPDLSCs.

Runx2 is a widely studied gene that has been identified as a key osteogenic gene in various cells. The protein level of Runx2 was increased after osteogenic differentiation in pPDLSCs (Figure 6(a)). Moreover, the mRNA level of Runx2 increased with osteogenic induction (Figure 6(b)). The miR-30 family has been reported to be extremely important in osteogenic differentiation, and some members of the family can downregulate the key osteogenic gene Runx2. Therefore, to explore the relationship between miR-30b-3p and Runx2, we predicted their binding sites on MicroInspector online software. We found that a previous study demonstrated the binding of miR-30b-3p to Runx2 through dual-luciferase reporter assay [43], qRT-PCR, and western blotting [44, 45]. Moreover, the miR-30b-3p mimic negatively regulated Runx2 mRNA and protein levels, while the miR-30b-3p inhibitor positively regulated Runx2 mRNA and protein levels (Figures 6(c) and 6(d)). Thus, all the above indicated that miR-30b-3p inhibits Runx2, even without osteogenic induction. Furthermore, in the absence of osteogenic induction, the mRNA and protein levels of Runx2 were increased after overexpression of lncRNA CUTALP, while the mRNA and protein levels of Runx2 were decreased after knockdown of lncRNA CUTALP (Figures 6(e) and 6(f)). To further clarify the relationship between lncRNA, miR-30b-3p, and Runx2, we investigated the expression of Runx2 and found that it decreased after lncRNA CUTALP knockdown, but this change was reversed by the miR-30b-3p inhibitor (Figures 6(g) and 6(h)). To demonstrate the interaction `between METTL3 and lncRNA CUTALP, as well as the interaction between lncRNA CUTALP and miR-30b-3p, we carried IF and FISH experiments to observe the localization of METTL3 and lncRNA CUTALP in pPDLSCs. There was no significant difference in the localization of lncRNA CUTALP in the cytoplasm and nucleus. But, METTL3 was found to be slightly more localized in the cytoplasm than in the nucleus (Figure S9). Thus, lncRNA CUTALP modulates Runx2 through the regulation of miR-30b-3p. In summary, the METTL3/lncRNA CUTALP/miR-30b-3p/Runx2 axis promoted osteogenic differentiation of pPDLSCs (Figure 7).

## 4. Discussion

M6A modification has recently been studied extensively. Previous studies on m6A modification have mostly focused on mRNA. M6A modification is involved in the splicing of mRNA, regulating the nuclear output of mRNA, regulating the translation of mRNA, and affecting the stability of mRNA [11]. For example, Wang et al. [46] found that METTL3-mediated m6A modification promotes angiogenesis and glycolysis in gastric cancer by enhancing HDGF mRNA stability. Sheng et al. [47] found that METTL3 regulates the translation efficiency of nuclear factor I-C (NFCI) in human dental pulp cells (hDPCs), thus affecting root formation. However, there are relatively few studies on the effect of METTL3-mediated m6A modification on lncRNAs. Several studies have been reported that METTL3-mediated m6A modification regulates the expression and stability of lncRNAs [48, 49]. The research on the regulation of lncRNAs by m6A modification mostly focuses on cancer, and there are few studies in other fields. There are two studies on the regulatory role of METTL3-mediated m6A modification of lncRNAs during osteogenic differentiation. Song et al. [23] found that METTL3 activated the MAPK signaling pathway by regulating the m6A modification level and expression level of lncRNA (RP11-44N12.5), thus promoting osteogenic differentiation of hASCs. Yuan et al. [50] found that the METTL3/lncRNA XIST/miR-302a-3p/USP8 axis promotes osteogenic differentiation of ossification of the posterior longitudinal ligament. Our study focused on the role of METTL3-mediated m6A modification of lncRNAs in the osteogenic differentiation of pPDLSCs. We verified that the METTL3/lncRNA CUTALP/miR-30b-3p/Runx2 axis enhances the osteogenic differentiation of pPDLSCs, including upregulated expression of osteogenic genes and osteogenic proteins and enhanced ALP staining and ARS. Another study demonstrated that METTL3-mediated m6A positively regulates Runx2 by regulating miR-320, thus regulating the osteogenic differentiation of bone mesenchymal stem cells (BMSCs) [51]. Similar to previous studies, we also proved that METTL3-mediated m6A modification is a positive regulator of osteogenic differentiation. In the field of oral cavity, the research on m6A methylation modification is increasing gradually. In the field of oral cavity, the research on m6A methylation modification is increasing gradually. Most of them are concentrated in oral squamous cell carcinoma; for example, METTL3 depletion profoundly reduced cell proliferation, cell migration, oncogenicity, and cisplatin resistance of arecoline-exposed OSCC cells [52]. In addition, other studies have demonstrated that METTL3 and METTL14 are overexpressed in OSCC and have found that overexpressed METTL3 or METTL14 is an independent prognostic factor for short overall survival in OSCC patients [53]. However, m6A methylation modification in oral nonneoplastic diseases has been relatively poorly studied. Luo et al. [54] found that METTL3 inhibition comprised DPSC (dental pulp stem cells) differentiation, and METTL3 overexpression facilitated DPSC mineralization. Inhibition of ALKBH5 or FTO promotes resistance of human oral epithelial cells to herpes simplex virus type I infection by increasing m6A methylation modification [55].

LncRNA is a kind of ncRNA that mostly regulates downstream target genes by competitively binding miRNA. Through m6A microarray analysis, we identified the downstream molecule regulated by the m6A-related protein METTL3 as NR_024408 and named it lncRNA CUTALP. We verified by MeRIP-qPCR that the m6A modification level of lncRNA CUTALP was consistent with that of the microarray, and its expression was positively correlated with Runx2 at different time points of osteogenic differentiation of pPDLSCs. MiR-30b-3p is a member of the miR-30 family, which has been widely reported to be involved in the regulation of osteogenic differentiation of various types of cells [44]. Tu et al. [43] found that the lncRNA CALB2/miR-30b-3p/Runx2 axis is a regulatory mechanism of odontogenic differentiation of hDPSCs. However, the function of miR-30b-3p in PDLSCs remains to be further investigated. Our study demonstrated that lncRNA CUTALP/miR-30b-3p/Runx2 regulates the osteogenic differentiation of pPDLSCs. MiR-30b-3p, similar to other members of the family, inhibits Runx2 expression and thus decreases osteogenic differentiation.

The osteogenic differentiation of pPDLSCs has been widely concerned by scholars, and it has been reported that proteins, lncRNAs, miRNAs, and epigenetic modifications all regulate this process. Most of the previous studies on pPDLSCs linked protein‒mRNA or lncRNA‒miRNA–mRNA, but few studies linked epigenetic modification with lncRNA. We demonstrated for the first time in pPDLSCs that the METTL3/lncRNA CUTALP/miR-30b-3p/Runx2 axis is a regulatory network of osteogenic differentiation. This discovery provides a new entry point for the osteogenic differentiation of pPDLSCs. However, our research is still limited to the traditional mechanism of lncRNA, which is not innovative. The mechanisms of m6A-modified proteins are extensive. For example, METTL3 interacts with m6A readers to promote gene expression. METTL3 assists YTHDF3 in promoting YAP expression and recruits the YTHDF1/3 and eIF3b complexes to enhance YAP translation [48]. METTL3 regulates Runx2 expression not only in pPDLSCs. Tian et al. [56] found that METTL3 knockdown resulted in decreased expression of osteogenic-associated proteins, including Runx2 and Osterix, in BMSCs. However, the specific pathway by which METTL3 regulates Runx2 remains unclear. We wondered if METTL3 could directly regulate Runx2 expression by recruiting complexes or altering mRNA structure. We will also focus on this issue for further exploration in the future. If this conjecture is confirmed, the osteogenic differentiation of periodontitis will be further elucidated. Our research also lacks animal models, and cell experiments are quite different from the in vivo environment.

Therefore, we will further conduct animal experiments to explore the regulatory effect of METTL3 on osteogenic differentiation in the periodontitis microenvironment.

## 5. Conclusion

In conclusion, we confirmed that METTL3-mediated m6A modification promotes the expression and stability of lncRNA CUTALP in pPDLSCs, thus inhibiting miR-30b-3p and ultimately regulating the expression of Runx2, a key osteogenic gene. Our study will help elucidate osteogenic differentiation and complement the mechanism of bone formation in periodontitis.

## Figures and Tables

**Table 1:** Comparison between the array data and MeRIP-qPCR data.

LncRNA name	Array data		MeRIP-qPCR data	
	Fold change	*P*-value	Fold change	*P*-value
ENST00000613868	9.5566 (up)	0.0034	1.3500 (up)	0.0242
ENST00000414547	2.6127 (up)	0.0364	0.9787 (down)	0.7330
ENST00000550404	2.3354 (up)	0.0116	1.2627 (up)	0.0036
ENST00000630728	0.0787 (down)	0.0184	0.7998 (down)	0.0237
ENST00000439875	0.1181 (down)	0.0101	0.9693 (down)	0.6388
LncRNA CUTALP (transcript ID: NR_024408)	0.1526 (down)	0.0177	0.8950 (down)	0.0006

## Data Availability

All data generated or analyzed during this study are included in this article.

## Abbreviations

hPDLSCs: Human periodontal mesenchymal stem cells
pPDLSCs: Periodontal mesenchymal stem cells in patients with periodontitis
MSCs: Mesenchymal stem cells
MeRIP-qPCR: Methylated RNA immunoprecipitation-PCR
qRT-PCR: Quantitative real-time polymerase chain reaction
RIP: RNA-binding protein immunoprecipitation
FISH: Fluorescence in situ hybridization
IF: Immunofluorescence
ALP: Alkaline phosphatase
ARS: Alizarin red staining
m6A: N6-methyladenosine
mESCs: Mouse embryonic stem cells
HPDL: Human periodontal ligament
hASCs: Human adipose-derived stem cells
hBMSCs: Human bone marrow-derived mesenchymal stem cells
Osx: Osterix
NFCI: Nuclear factor I-C
hDPCs: Human dental pulp cells
hDPSCs: Human dental pulp stem cells
BMSCs: Bone mesenchymal stem cells.

## Supplementary Materials

Supplementary Materials The supplementary materials include the sequences of miRNA mimic/inhibitor and lentivirus and transfection methods. Table S1 of supplementary materials lists the clinical information of the patients from whom the teeth came. Table S2 of supplementary materials includes all primer sequences of the research. Figures S1 and S2 show the transfection efficiency of lentivirus. Figure S3 shows the cell morphology. Figures S4, S5, S6, S7, and S8 are the quantitative analysis diagrams for ALP and ARS assay. Figure S9 shows the localization of METTL3 and lncRNA CUTALP detected by IF and FISH assay in pPDLSCs. Bar = 10 *µ*m.
