# Supplementary material for: METTL3-Mediated m6A Modification Regulates the Osteogenic Differentiation through LncRNA CUTALP in Periodontal Mesenchymal Stem Cells of Periodontitis Patients
Source: Stem Cells Int. 2024 Jan 19;2024:3361794. doi: 10.1155/2024/3361794 (PMC10817817; doi:10.1155/2024/3361794)
Supplement: Supplementary Materials — The supplementary materials include the sequences of miRNA mimic/inhibitor and lentivirus and transfection methods. Table S1 of supplementary materials lists the clinical information of the patients from whom the teeth came. Table S2 of supplementary materials includes all primer sequences of the research. Figures S1 and S2 show the transfection efficiency of lentivirus. Figure S3 shows the cell morphology. Figures S4, S5, S6, S7, and S8 are the quantitative analysis diagrams for ALP and ARS assay. Figure S9 shows the localization of METTL3 and lncRNA CUTALP detected by IF and FISH assay in pPDLSCs. Bar = 10 µm. [file 3361794.f1.docx]

**METTL3-Mediated m6A Modification Regulates the Osteogenic Differentiation through LncRNA CUTALP in Periodontal Mesenchymal Stem Cells of Periodontitis Patients**

**Authors:** Xin Chen^1^, Yuan Qin^1*^, Xian Wang^1^, Hao Lei^2^, Xiaochen Zhang^1^, Houzhuo Luo^1^, Changgang Guo^1^, Weifu Sun^1^, Shishu Fang^1^, Wen Qin^1^, Zuolin Jin^1^

**Affiliations:**

^1^State Key Laboratory of Military Stomatology & National Clinical Research Center for Oral Diseases & Shaanxi Clinical Research Center for Oral Diseases, Department of Orthodontics, School of Stomatology, Air Force Medical University, Xi’an, 710032, China.

^2^Department of Dermatology, the First Affiliated Hospital of Xi'an Jiaotong University, Xi'an, 730070, China.

**Corresponding author:** Zuolin Jin; Email: zuolinj@163.com

**Co-corresponding author:** Wen Qin; Email: qinwen0916@126.com

**Supplementary Material**

**Transfection of miRNA mimic and inhibitor.**

All sequences are as following:

miR-30b-3p mimic (sense, 5’-CUGGGAGGUGGAUGUUUACUUC-3’; antisense, 5’-AGUAAACAUCCACCUCCCAGUU-3’), control (sense, 5’-UUCUCCGAACGUGUCACGUTT-3’; antisense, 5’-ACGUGACACGUUCGGAGAATT-3’), miR-30b-3p inhibitor (5’-GAAGUAAACAUCCACCUCCCAG-3’), and inhibitor control (5’-CAGUACUUUUGUGUAGUACAA-3’). Target cells were transfected with GP-transfect-mate (GenePharma, Shanghai, China) according to the instructions.

**Construction and transduction of** **lentivirus.**

Lentiviral production is produced by GenePharma (Shanghai, China). The lentiviral vector overexpressing METTL3 and lncRNA CUTALP was METTL3-Homo and lncRNA CUTALP-Homo. The lentiviral vector that knockdown METTL3 and lncRNA CUTALP was shMETTL3-1/2/3 and shlncRNA CUTALP-1/2/3. LV-NC5 and LV-NC3 were the negative controls of overexpression and knockdown vector. Lentiviral vectors were added to the minimum necessary medium according to the instructions, and Ploybrene (GenePharma, Shanghai, China) was co-transfected to enhance transfection efficiency. All sequences are as follows:

shlncRNA CUTALP-1: GCCCTCATGACCTTGTTTA,

shlncRNA CUTALP-2: GGCGGATCATTAGGTCAAA,

shlncRNA CUTALP-3: AGGGCATGAGGATGGTTATAT;

shMETTL3-1: GCTGCACTTCAGACGAATTAT,

shMETTL3-2: GCTACCTGGACGTCAGTATCT,

shMETTL3-3: GCTCAACATACCCGTACTACA,

LV-NC3: TTCTCCGAACGTGTCACGT,

LV-NC5 is an empty vector without sequence.

**Table S1:** The clinical information of the patients.

| **Patient** | **Age** | **Cell** | **Dental** | **Periodontal pocket (mm)** |
| --- | --- | --- | --- | --- |
| 1 | 31 | hPDLSC | premolar | 1.00 |
| 2 | 33 | hPDLSC | premolar | 1.50 |
| 3 | 35 | hPDLSC | premolar | 0.80 |
| 4 | 37 | hPDLSC | third molar | 0.00 |
| 5 | 37 | hPDLSC | third molar | 0.50 |
| 6 | 37 | hPDLSC | third molar | 0.80 |
| 7 | 37 | hPDLSC | premolar | 1.25 |
| 8 | 40 | hPDLSC | third molar | 0.50 |
| 9 | 36 | pPDLSC | premolar | 5.20 |
| 10 | 38 | pPDLSC | premolar | 5.50 |
| 11 | 38 | pPDLSC | third molar | 6.10 |
| 12 | 39 | pPDLSC | first molar | 5.80 |
| 13 | 39 | pPDLSC | second molar | 5.05 |
| 14 | 40 | pPDLSC | premolar | 6.55 |
| 15 | 40 | pPDLSC | premolar | 5.85 |
| 16 | 41 | pPDLSC | first molar | 6.05 |

**Table S2:** The sequences of Primers.

| **Target Genes** | **Primer Sequences (5’to 3’)** |
| --- | --- |
| LncRNA CUTALP-F  (Transcript ID: NR_024408) | CTGCCGTAGCCAACAATGAAG |
| LncRNA CUTALP-R  (Transcript ID: NR_024408) | ACCGTGCCTGGGAAACAATA |
| Runx2-F | CCCGTGGCCTTCAAGGT |
| Runx2-R | CGTTACCCGCCATGACAGTA |
| ALP-F | GGACCATTCCCACGTCTTCAC |
| ALP-R | CCTTGTAGCCAGGCCCATTG |
| Col1-F | CCAGAAGAACTGGTACATCAGCAA |
| Col1-R | CGCCATACTCGAACTGGAATC |
| β-actin-F | TGGCACCCAGCACAATGAA |
| β-actin-R | CTAAGTCATAGTCCGCCTAGAAGCA |
| METTL3-F | TGGGGGTATGAACGGGTAGA |
| METTL3-R | CCTTTGACACCAACCAAGCAG |
| METTL14-F | CCCATGTACTTACAAGCCGATAT |
| METTL14-R | ATTAGCAGTGATGCCAGTTTCTC |
| ALKBH5-F | TGTGCTTCGGCTGCAAGTTC |
| ALKBH5-R | CCTGAGGCCGTATGCAGTGA |
| FTO-F | CAGGGTTGGGATGGGTTCA |
| FTO-R | CGTTGTATGCTGCTCTGCTCTTA |
| U6-F | GGAACGATACAGAGAAGATTAGC |
| U6-R | TGGAACGCTTCACGAATTTGCG |
| hsa-miR-30b-3p | CTGGGAGGTGGATGTTTACT |
| ENST00000613868-F | TGATGGAGAAGCCGCTGTT |
| ENST00000613868-R | TTCCGTCCCATAATCAACTGTCTT |
| ENST00000414547-F | TCGAATGCCTGCACTTGGA |
| ENST00000414547-R | GCTGGCAGTGGTCAGACT |
| ENST00000550404-F | TAGCAACCACCAAGACACAGT |
| ENST00000550404-R | AGATGATAGAACACCTGGCTTCC |
| ENST00000630728-F | GAGGTTCAAGACAGCAGTGAGA |
| ENST00000630728-R | AAGGGAGAGCAAATCAAAGGAAAC |
| ENST00000439875-F | TGCCACCGTATTCTTCATTCCT |
| ENST00000439875-R | CCCACATCTAACCCTTCCTCTG |

**Supplementary Figure**


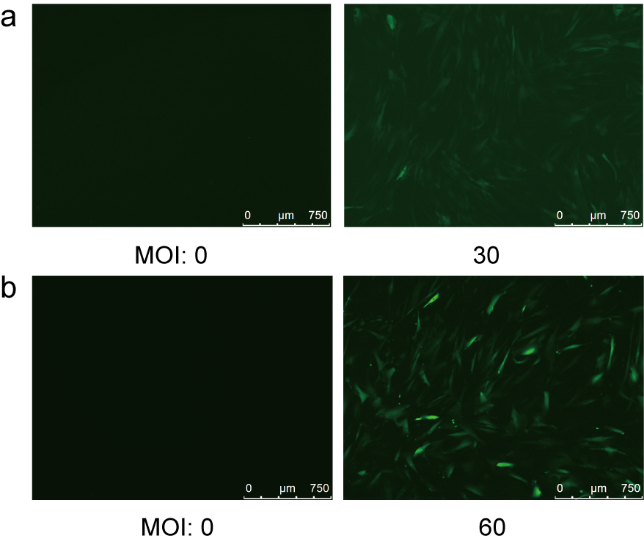


Fig. S1 The expression of GFP in each group after 72h transfection with lentivirus was observed by fluorescence microscope. Scale bar = 750 μm. (a) METTL3-overexpression. MOI = 30. (b) METTL3-knockdown. MOI = 60.


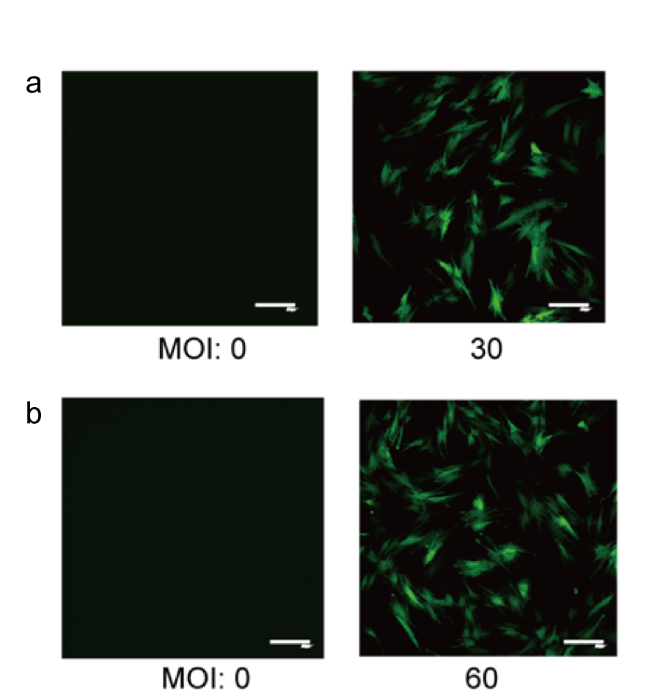


Fig. S2 The expression of GFP in each group after 72h transfection with lentivirus was observed by confocal microscope. Scale bar = 200 μm. (a) lncRNA CUTALP-overexpression. MOI = 30. (b) lncRNA CUTALP -knockdown. MOI = 60.


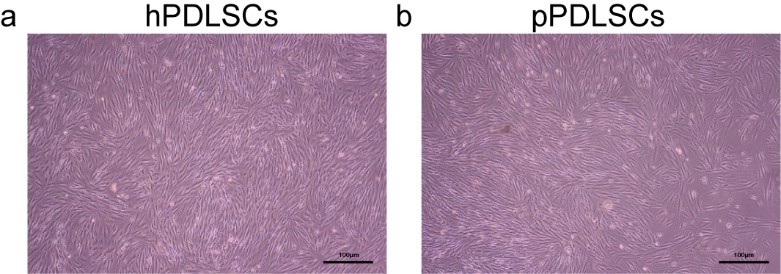


Fig. S3 The morphological figures under the cell microscope. (a) The third-generation hPDLSCs. (b) The third-generation pPDLSCs. Bar = 100μm.


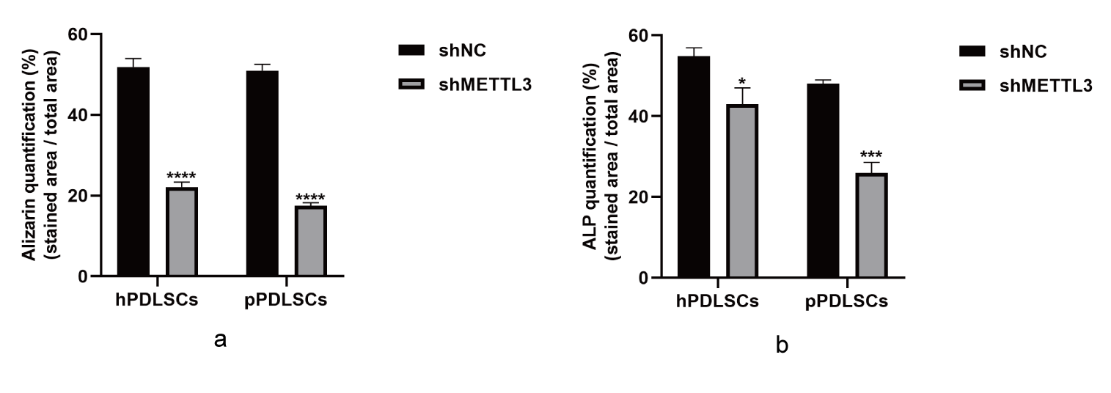


Fig. S4 The quantitative analysis diagram for ARS staining (a) and ALP staining (b) at 21 and 7 days of osteogenic induction after METTL3 knockdown.


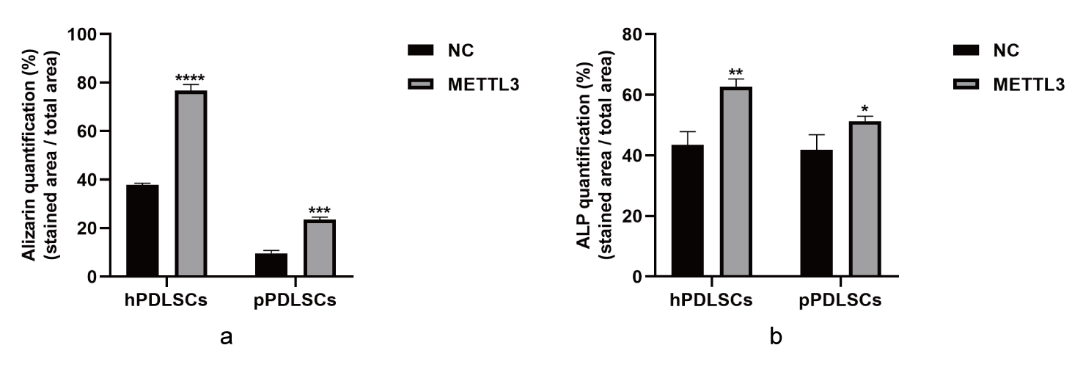


Fig. S5 The quantitative analysis diagram for ARS staining (a) and ALP staining (b) at 21 and 7 days of osteogenic induction after METTL3 overexpression.


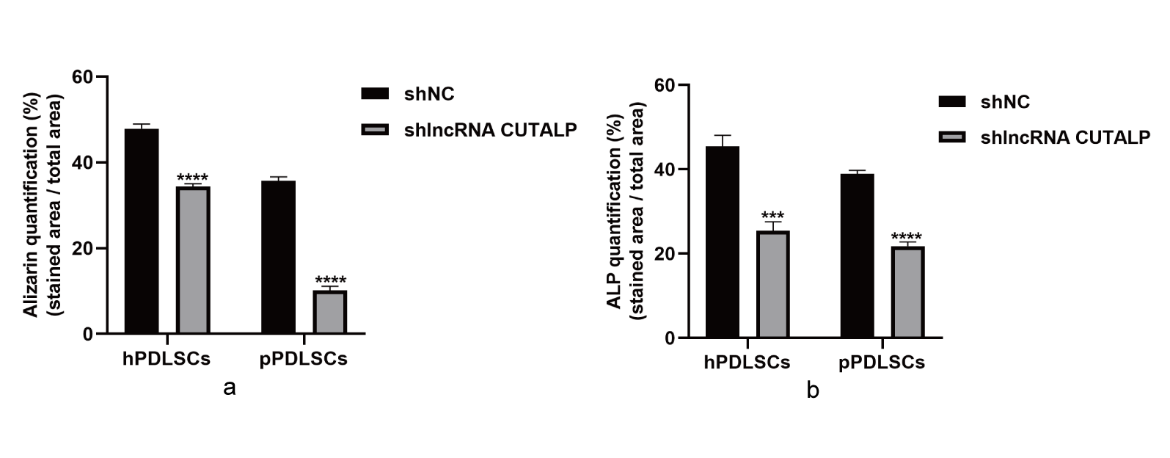


Fig. S6 The quantitative analysis diagram for ARS staining (a) and ALP staining (b) at 21 and 7 days of osteogenic induction after lncRNA CUTALP knockdown.


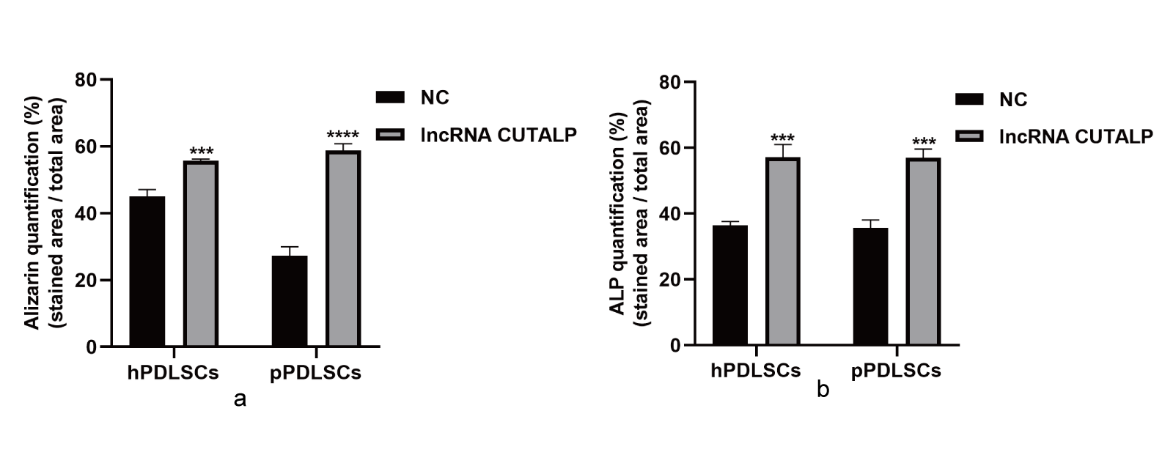


Fig. S7 The quantitative analysis diagram for ARS staining (a) and ALP staining (b) at 21 and 7 days of osteogenic induction after lncRNA CUTALP overexpression.


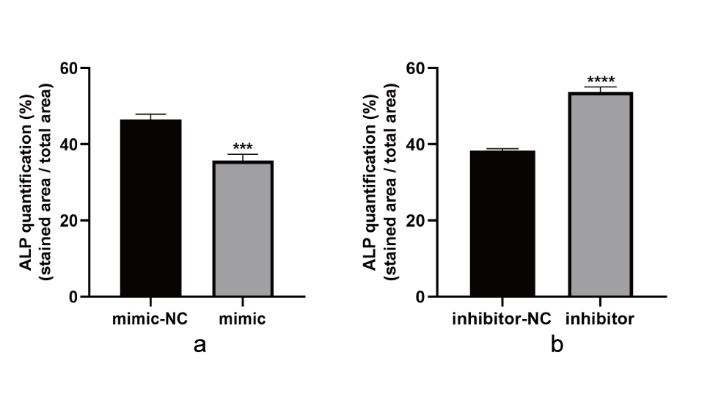


Fig. S8 The quantitative analysis diagram for ALP staining at 7 days of osteogenic induction after miR-30b-3p mimic (a) or inhibitor (b) transfected.


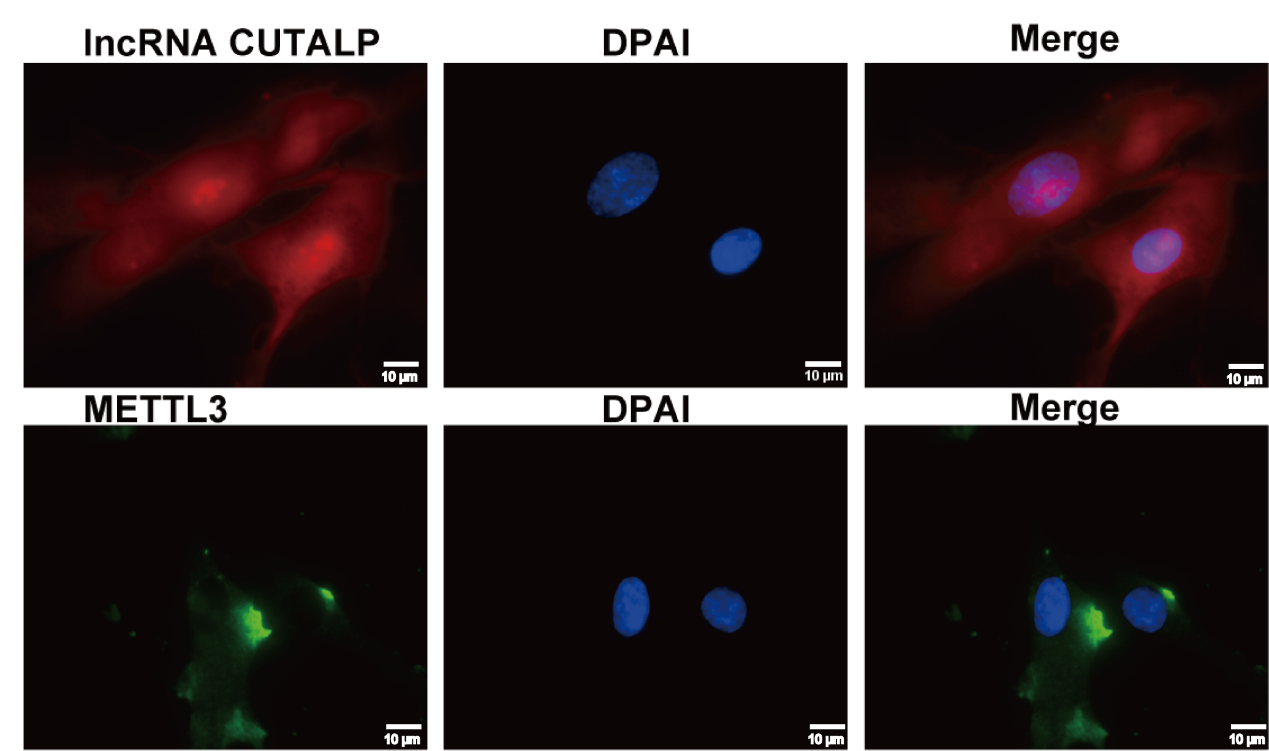


Fig. S9 The localization of METTL3 and lncRNA CUTALP detected by IF and FISH assay in pPDLSCs. bar = 10 μm.
